# Blockage of ERCC6 Alleviates Spinal Cord Injury Through Weakening Apoptosis, Inflammation, Senescence, and Oxidative Stress

**DOI:** 10.3389/fmolb.2022.853654

**Published:** 2022-02-22

**Authors:** Peng Zou, Xiaoping Zhang, Rui Zhang, Xin Chai, Yuanting Zhao, Erliang Li, Qian Zhang, Rongbao Yan, Junsong Yang, Bo Liao

**Affiliations:** ^1^ Department of Spinal Surgery, Tangdu Hospital, Second Affiliated Hospital of Air Force Military Medical University, Xi’an, China; ^2^ Department of Spinal Surgery, Honghui Hospital, Xi’an Jiaotong University, Xi’an, China

**Keywords:** spinal cord injury, ERCC6, apoptosis, inflammation, senescence, oxidative stress

## Abstract

**Objective:** Spinal cord injury (SCI) is a devastating disease resulting in lifelong disability, but the molecular mechanism remains unclear. Our study was designed to observe the role of excision repair cross-complementing group 6 (ERCC6) following SCI and to determine the underlying mechanism.

**Methods:** SCI mouse models and LPS-induced microglia cell models were established. ERCC6 expression was blocked by ERCC6-siRNA-carrying lentivirus. Nissl staining was utilized for detecting neuronal damage, and apoptosis was analyzed with TUNEL and Western blotting (apoptotic markers). Immunofluorescence was used for measuring macrophage markers (CD68 and F4/80) and astrocyte and microglia markers (GFAP and Iba-1). Pro-inflammatory cytokines (TNF-α, IL-1β, and IL-6) were measured *via* ELISA. Senescent cells were estimated *via* SA-β-Gal staining as well as Western blot (senescent markers p21 and p27). Oxidative stress was investigated by detecting the expression of 4-HNE, Nrf2, and Keap1, and intracellular ROS levels.

**Results:** ERCC6 expression was remarkably upregulated both in the spinal cord of SCI mice and LPS-induced microglia cells. ERCC6 deficiency alleviated neuronal damage and apoptosis. Macrophage infiltration and inflammatory response were suppressed by si-ERCC6 treatment. Moreover, ERCC6 blockage ameliorated astrocyte and microglia activation and cell senescence in the damaged spinal cord. Excessive oxidative stress was significantly decreased by ERCC6 knockdown in SCI.

**Conclusion:** Collectively, ERCC6 exerts crucial functions in mediating physiological processes (apoptosis, inflammation, senescence, and oxidative stress), implying that ERCC6 might act as a prospective therapeutic target against SCI.

## Introduction

Spinal cord injury (SCI), a highly disabling neurological disorder, usually leads to permanent motor and sensory dysfunctions, affecting approximately 250,000–500,000 individuals each year (Zhou et al., 2020). SCI is characterized by the lesion cores or fibrotic scars without viable neural tissues, the scars of astrocytes around the lesion cores, and the area of surviving neural tissues with limited functions and functional plasticity (Zou, 2021). Despite the structural support provided by the lesion scars, there is a suppressive environment for the regeneration of severed axons, thereby suppressing the re-debilitation of the original target (Kuboyama et al., 2021). SCI is also characterized by astrocyte and infiltrating macrophage activation in the spinal cord (Van Broeckhoven et al., 2021). Following damage, the blood–spinal cord barrier is destroyed. Despite gradual recovery, it will be damaged for a long time, promoting immune cell extravasation as well as chronic inflammation (Zrzavy et al., 2021). Due to complex pathophysiology (apoptosis, inflammation, senescence, oxidative stress, etc.), the therapeutic options of SCI are limited (Xue Wang et al., 2021). At present, several therapeutic interventions have been applied for SCI, such as high-dose methylprednisolone, ganglioside, and immunoglobulin G, which show favorable clinical benefits for a subset of patients (Wu et al., 2021). Nevertheless, these drugs cannot improve recovery after SCI to a large extent.

SCI involves primary and secondary damage (Hough et al., 2021). Primary injury is usually triggered by mechanical damage of the spinal cord, while secondary injury can be caused by the delayed sequences of complex biochemical as well as cell processes (Feng et al., 2021). Altogether, primary and secondary injuries result in irreversible neuronal injury, eventually culminating in unfavorable functional recovery after SCI (Zhang et al., 2021). Hence, to prevent irreversible injury by targeting specific molecules is a feasible therapeutic regimen against SCI. Excision repair cross-complementing group 6 (ERCC6, as shown as CSB), a DNA-binding protein, is of importance for transcription-coupled excision repair as well as DNA repair processes (Takahashi et al., 2019). This protein possesses ATP-stimulated ATPase activity as well as interacts with transcription and excision repair proteins, thereby promoting the formation of complexes at DNA repair sites (Takahashi et al., 2019). ERCC6 expression is frequently upregulated in cancer cells, favoring cancer cell proliferation while suppressing apoptosis, which has been recognized as a promising actionable target for cancer treatment (Proietti-De-Santis et al., 2018). Moreover, ERCC6 is involved in the pathogenesis of age-related diseases. For instance, ERCC6 may disrupt autophagic flux in age-related cataract *via* binding to VCP (Cao et al., 2021). Ultraviolet-B-induced ERCC6 inhibition contributes to age-related nuclear cataract (Wang et al., 2016). However, the role of ERCC6 in SCI remains unknown. Herein, we investigated whether ERCC6 was highly expressed following SCI and targeting ERCC6 alleviated apoptosis, inflammation, senescence, and oxidative stress both in the SCI mouse and cellular models. Hence, ERCC6 might become a therapeutic target against SCI.

## Materials and Methods

### Animals and Groups

All experimental protocols gained the approval of the Animal Research Committee of Tangdu Hospital, Second Affiliated Hospital of Air Force Military Medical University (KY-2020015). C57BL/6 mice (8–12 weeks old; 20–25 g) purchased from Changzhou Cavans Experimental Animal Co., Ltd. (China) were cared for strictly following the ethical guidelines on animal experimentation of Laboratory Animals of China National Institutes of Health. All mice were housed in a well-ventilated environment with a 12-h light–dark cycle, at 23 ± 2°C and 70 ± 10% humidity, with free food and water. They were randomized into control group, sham group, SCI-Day 3 group (SCI on day 3), SCI-Day 7 group (SCI on day 7), SCI-Day 14 group (SCI on day 14), and SCI-Day 28 group (SCI on day 28), with eight mice in each group. Abovementioned mice did not receive any medication. Other mice were randomly separated into control group, sham group, SCI + si-NC group, and SCI + si-ERCC6 group, with eight mice in each group.

### Establishment of SCI Mouse Models and Treatment

C57BL/6 mice were anesthetized by intraperitoneally injecting sodium pentobarbital (30 mg/kg). The limbs were fixed and the chest was raised with cotton pads. All animals were placed on a constant heating pad for maintaining at 37°C throughout the operation. The dorsal skin tissues were opened along the spinal column, and the muscles were peeled off layer by layer, and the T9 and T10 segments of the spine were located. Under an operating microscope, laminectomy was carried out at the T9 and T10 levels for exposing the surface of the dorsal cord without damaging the dura mater. For the SCI group, a 10 g weight was dropped from 1.5 cm to the exposed spinal cord for inducing a SCI contusion and for keeping the dura mater unbroken. Thereafter, the muscle as well as skin was sutured layer by layer utilizing 4–0 silks and needles. Following SCI, the mice were free to drink and eat, and the breeding environment was kept at a constant temperature of 22°C and a constant humidity of 30–50%. The criteria for successful modeling were as follows: spinal cord tissues of mice presented swelling, edema, and bleeding; hind limbs were paralyzed; and the mice were unable to urinate or defecate spontaneously. Manual bladder emptying was conducted 3 times each day until the voluntary urination was restored. The mice were given intraperitoneal injection of penicillin (0.2 ml/kg) for three consecutive days to prevent infection. The control group did not undergo any treatment. For the sham group, only after the spinal cord was exposed, the incision was closed layer by layer in mice. On 3, 7, 14, 21, and 28 days following SCI, animals were euthanized and spinal cord tissues were collected.

### Animal Treatment

For the evaluation of the functions of ERCC6 during spinal cord damage, siRNAs against ERCC6 (si-ERCC6) were cloned into LV-3 lentivirus vectors. The LV-3 vectors inserted with non-specific siRNAs were utilized as negative control (si-NC). In the SCI + si-NC group and SCI + si-ERCC6 group, the mice received SCI treatment, and were injected with si-NC or si-ERCC6 (10^5^ plaque-forming units) in the intrathecal space utilizing glass micropipettes following SCI. Following 28 days, mice were euthanized by intraperitoneally injecting pentobarbital sodium (200 mg ⁄ kg). Euthanasia was judged by complete cessation of heartbeat, breathing, and loss of reflexes. Then, spinal cord tissues were collected.

### Cell Culture

The murine BV2 microglial cell line (ATCC, United States) was grown in DMEM plus 10% FBS and 1% penicillin/streptomycin in a 5% CO_2_ incubator at 37 °C. Until the confluence reached about 80%, the cells were digested by trypsin and passaged for subsequent experiments. Lipopolysaccharide (LPS) was obtained from Sigma-Aldrich (United States). BV2 cells were exposed to LPS (0, 25, 50, and 100 ng/ml) lasting 24 h.

### Transfection of Small Interfering RNAs

SiRNAs against ERCC6 (si-ERCC6) and negative control (si-NC) were designed and synthesized by Generay Biotech (Shanghai, China). After treatment with LPS for 24 h, transfection of siRNAs was implemented in accordance with the manufacturer’s instructions. In brief, the Lipofectamine 3,000 transfection reagent (Beyotime, China) and siRNAs were mixed together in Opti-MEM. Thereafter, the cell culture medium was exchanged with Opti-MEM lasting 6 h. The cells were exchanged with the normal medium and continued to culture, lasting 48 h.

### Real-Time Quantitative PCR

Total RNA extracts were conducted utilizing TRIzol reagent. 5 μg RNA was utilized for synthesizing cDNA. RT-qPCR was implemented on SYBR Green (Sigma-Aldrich, United States). cDNA was amplified on the 7,500 fast RT-PCR system (ABI, United States). The relative mRNA expression was normalized to the housekeeping gene GAPDH with 2^−ΔΔCT^ approach. The oligonucleotide primers were as follows: ERCC6, 5′-ATG​TTC​CAC​GAG​GAA​GTT​CCC-3’ (forward) and 5′-GCC​CAA​CTG​GCA​TGT​CTT​TG-3’ (reverse), and GAPDH, 5′-AGG​TCG​GTG​TGA​ACG​GAT​TTG-3’ (forward) and 5′-GGG​GTC​GTT​GAT​GGC​AAC​A-3’ (reverse).

### Western Blotting

Extraction of total protein was conducted utilizing QIAzol™ lysis reagent (Qiagen, CA). 40 μg protein was subjected to SDS–PAGE as well as transferred onto PVDF membranes (Millipore, MA). Thereafter, the blots were blocked with 5% blotting grade milk lasting 1 h and were incubated with a primary antibody against ERCC6 (#24291-1-AP; 1:500; Proteintech, Wuhan, China), GAPDH (#10494-1-AP; 1:10000; Proteintech), Bax (#60267-1-Ig; 1:5000; Proteintech), cleaved caspase-3 (#19677-1-AP; 1:500; Proteintech), Bcl-2 (#26593-1-AP; 1:1000; Proteintech), p21 (#10355-1-AP; 1:1000; Proteintech), p27 (#25614-1-AP; 1:1000; Proteintech), 4-HNE (#ab46545; 1:3000; Abcam, United States), and Nrf2 (#16396-1-AP; 1:500; Proteintech) or Keap1 (#10503-2-AP; 1:2000; Proteintech) overnight at 4°C. The following day, the blots were incubated in horseradish peroxidase-conjugated goat anti-rabbit (#ab7090; 1:5000; Abcam) or anti-mouse secondary antibody (#ab7063; 1:5000; Abcam) lasting 1 h at room temperature. Thereafter, the blots were developed with enhanced chemiluminescence. The gray value was quantified *via* ImageJ software.

### Immunofluorescence Staining

The spinal cord close to the lesion epicenter was fixed by 4% paraformaldehyde, embedded in paraffin, and cut into 4-μm-thick sections. The spinal cord sections were permeabilized and sealed utilizing PBST with 1% BSA lasting 1 h at room temperature. Thereafter, the sections were incubated with the primary antibody against ERCC6 (#24291-1-AP; 1:100; Proteintech), CD68 (#CL594-25747; 1:100; Proteintech), or F4/80 (#ab6640; 1:100; Abcam) overnight at 4 °C. After rinsing with PBS, the slices were incubated by the secondary antibody lasting 1 h at room temperature. The nuclei were stained utilizing DAPI, and the slices were photographed under a fluorescence microscope (Olympus, Japan).

### Nissl Staining

Nissl staining (Solarbio, China) was conducted for detecting neuronal damage. The spinal cord sections were rinsed with PBS, and were maintained at 55 °C lasting 3 hours. Thereafter, the slices were placed into 0.9% crystal violet lasting 2 h at 37°C. Thereafter, slice dehydration was achieved in 70, 80, 90, and 100% ethanol lasting 5 minutes, followed by mounting utilizing neutral balsam. Investigation of images was implemented under a fluorescence microscope.

### Terminal-Deoxynucleoitidyl Transferase Mediated Nick End Labeling Staining

Apoptosis was measured utilizing the TUNEL kit (Solarbio, China). BV2 cells were planted onto glass coverslips in a 24 well-plate. These cells were fixed by 4% paraformaldehyde lasting 20 min at 37°C. Following permeabilization by 0.1% Triton X-100/PBS lasting 15 min, the cells were sealed by PBS with 5% BSA at room temperature for 1 hour. Thereafter, incubation of the spinal cord slices or cells with TUNEL solution was implemented at 4 °C overnight. TUNEL-positive cells under five random fields of view were estimated using ImageJ software.

### Enzyme-Linked Immunosorbent Assays

100 mg of the spinal cord was used for homogenate. Following centrifugation at 1,500 g lasting 15 min 4°C, the supernatant was harvested, and the levels of interleukin (IL)-1β (#SEKM-0002) and IL-6 (#SEKM-0007), and tumor necrosis factor-α (TNF-α; # SEKM-0034) in spinal cord tissues were measured *via* ELISA kits (Solarbio, China). The detection process was conducted in strict accordance with the corresponding kit instructions. The microplate reader was utilized for detecting the absorbance at 450 nm.

### Senescence-Associated β-Galactosidase Staining

SA-β-Gal staining (#C0602; Beyotime, China) was conducted in accordance with the manufacturer’s instruction. In brief, spinal cord tissues were washed with PBS as well as fixed lasting 15 min at room temperature. Thereafter, the tissues were incubated in the SA-β-gal staining solution overnight at 37°C and photographed under a light microscope (Olympus, Japan).

### Intracellular Reactive Oxygen Species Evaluation

The intracellular ROS was quantified utilizing ROS assay kits utilizing dichloro-dihydro-fluorescein diacetate (DCFH-DA; #287810; Sigma-Aldrich, United States) oxidized to fluorescent probes. BV2 cell line was stained by DCFH-DA following the manufacturer’s specifications. The intracellular ROS levels were determined in accordance with 488 nm excitation wavelength as well as 525 nm emission wavelength.

### Statistical Analysis

All analyses were implemented utilizing GraphPad Prism 8 software. One-way analysis of variance (ANOVA) was applied for comparisons among groups, with Tukey’s *post hoc* testing. Results were considered significant when *p*-value < 0.05. All quantitative data are displayed as mean ± standard deviation.

## Results

### ERCC6 Is Highly Expressed in SCI Mice and LPS-Induced Mouse Microglia Cells

**FIGURE 1:**
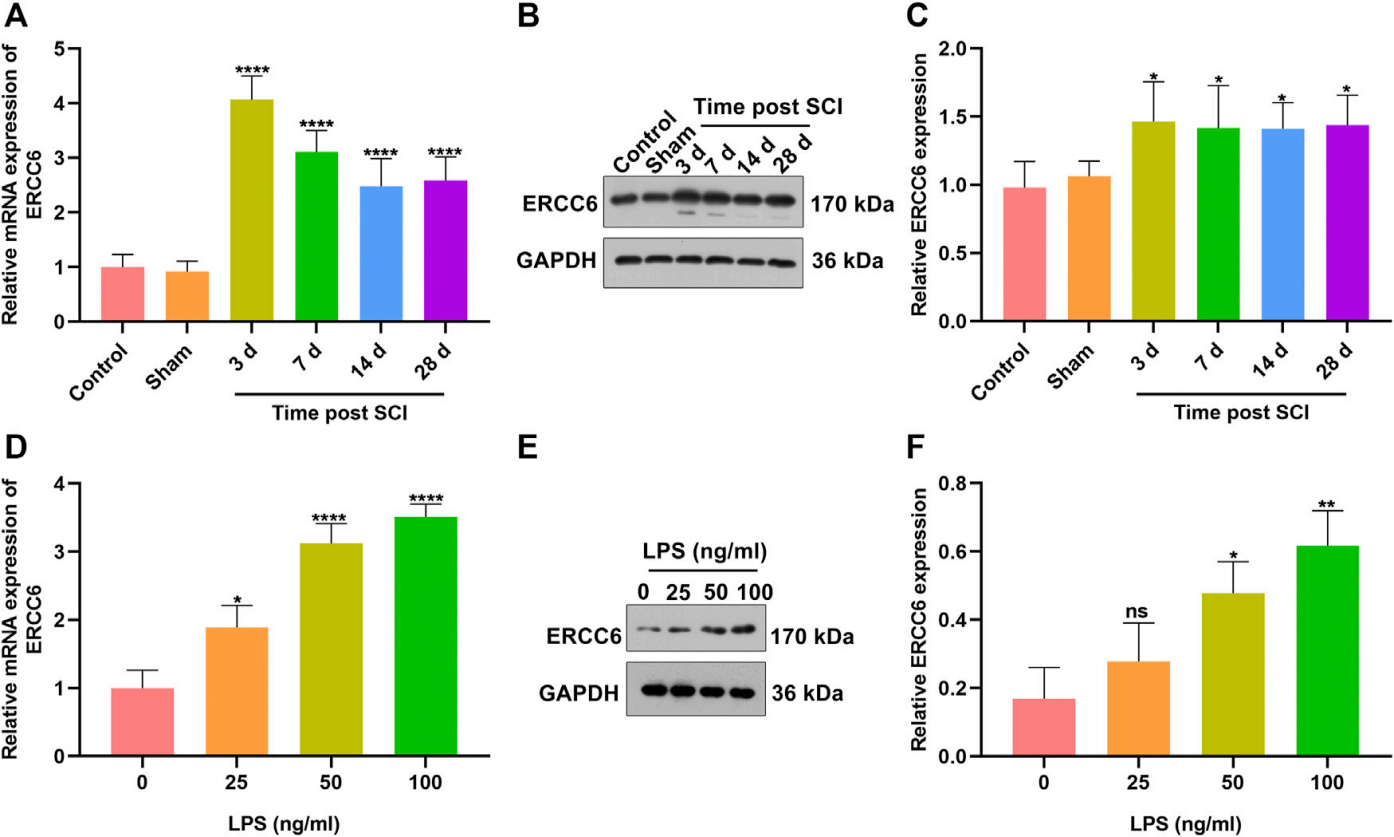
ERCC6 is highly expressed in SCI mice and LPS-induced mouse microglia cells. **(A)** RT-qPCR analysis of ERCC6 expression in the damaged spinal cord of mouse models on days 3, 7, 14, and 18 following ICI operations as well as control and sham operation mice (N = 8 mice in each group). **(B,C)** Western blotting analysis of ERCC6 expression in the damaged spinal cord of mouse models on days 3, 7, 14, and 18 following ICI operations as well as control and sham operation mice (N = 8 mice in each group). **(D)** RT-qPCR analysis of ERCC6 expression in BV2 mouse microglia cells exposed to distinct concentrations of LPS (N = 3 mice in each group). **(E,F)** Western blotting analysis of ERCC6 expression in BV2 mouse microglia cells under exposure to distinct concentrations of LPS (N = 3 mice in each group). *p*-values were calculated with the ANOVA test. Ns: no significance; *p-value < 0.05; **p-value < 0.01; ****p-value < 0.0001.

To investigate the role of ERCC6 during SCI, we established SCI mouse models and measured the ERCC6 expression alterations. In Figure 1A, ERCC6 presented significantly higher mRNA expression in damaged spinal cord on Days 3, 7, 14, and 18 following operations than sham operation mice. Similarly, ERCC6 protein expression was significantly higher in the spinal cord tissues of SCI mice on Days 3, 7, 14, and 18 after operations (Figures 1B,C). We also constructed LPS-induced BV2 mouse microglia cell models to simulate SCI. As LPS concentration increased, ERCC6 expression was significantly elevated in BV2 cells (Figures 1D–F). *In vitro* and *in vivo* evidence suggested that ERCC6 upregulation might enable to participate in SCI progression.

### ERCC6 Knockdown Alleviates Neuronal Damage in ICI Mice

**FIGURE 2:**
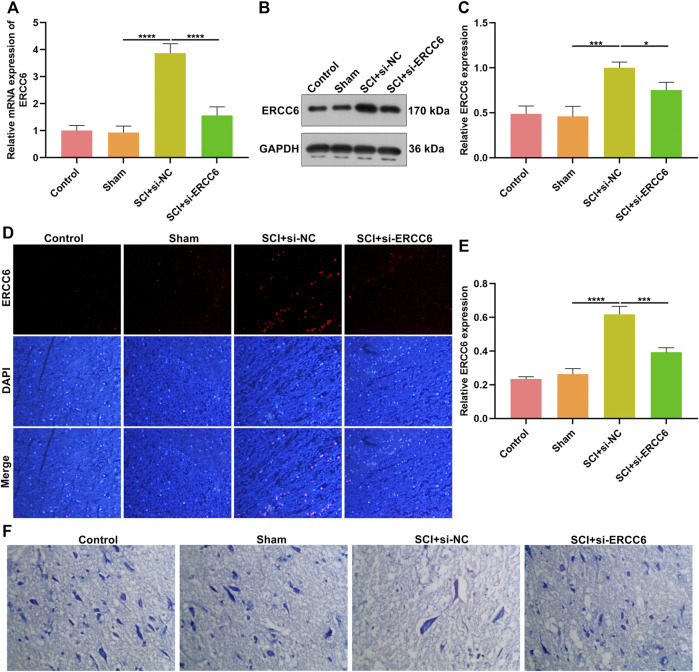
ERCC6 knockdown alleviates neuronal damage in the ICI mice. **(A)** RT-qPCR analysis of ERCC6 expression in the spinal cord of SCI mice injected with si-ERCC6 or si-NC as well as control and sham operation mice. **(B,C)** Western blotting analysis of ERCC6 expression in the damaged spinal cord of mouse models injected by si-ERCC6 or si-NC as well as control and sham operation mice. **(D,E)** Immunofluorescence staining of ERCC6 expression in the damaged spinal cord of mouse models injected by si-ERCC6 or si-NC as well as control and sham operation mice (scale bar, 50 μm). **(F)** Nissl staining of spinal cord sections of SCI mice injected with si-ERCC6 or si-NC as well as control and sham operation mice (scale bar, 50 μm; N = 8 mice in each group). *p*-values were calculated with the ANOVA test. *p-value < 0.05; ***p-value < 0.001; ****p-value < 0.0001.

We investigated whether ERCC6 deficiency enabled to alleviate SCI. Following injection with si-ERCC6 for 3 days, ERCC6 expression was measured in the spinal cord of SCI mice. As expected, ERCC6 expression was remarkably decreased in damaged spinal cord with si-ERCC6 treatment relative to those with si-NC treatment (Figures 2A–E). Nissl staining was conducted for detecting neuronal damage. In comparison with sham operation mice, the number of Nissl bodies in damaged spinal cord was significantly reduced (Figure 2F). Si-ERCC6 treatment remarkably increased the number of Nissl bodies in the spinal cord tissues of SCI mice. Hence, ERCC6 deficiency enabled to alleviate neuronal damage in ICI mice.

### ERCC6 Knockdown Decreases Apoptosis in Spinal Cord of ICI Mice and LPS-Induced Mouse Microglia Cells

**FIGURE 3:**
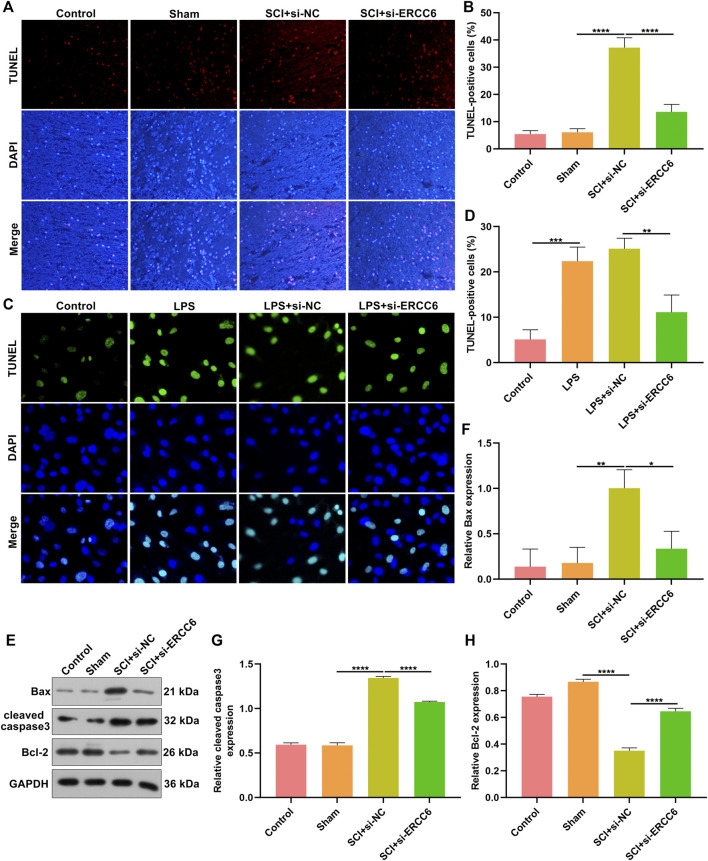
ERCC6 knockdown decreases apoptosis in the spinal cord of ICI mice and LPS-induced mouse microglia cells. **(A,B)** TUNEL staining of apoptotic levels in the damaged spinal cord of mouse models injected with si-ERCC6 or si-NC as well as control and sham operation mice (scale bar, 50 μm; N = 8 mice in each group). **(C,D)** TUNEL staining of apoptotic levels in LPS-exposed BV2 mouse microglia cells treated with si-ERCC6 or si-NC (scale bar, 50 μm; N = 3 mice in each group). **(E–H)** Western blotting analysis for Bax, cleaved caspase-3 as well as Bcl-2 in the spinal cord tissues of SCI mice injected with si-ERCC6 or si-NC as well as control and sham operation mice (N = 8 mice in each group). *p*-values were calculated with ANOVA test. *p-value < 0.05; **p-value < 0.01; ***p-value < 0.001; ****p-value < 0.0001.

TUNEL staining was conducted for investigating the apoptosis alterations in spinal cord tissues. As illustrated in Figures 3A,B, the apoptotic level was remarkedly elevated in the damaged spinal cord relative to sham operation mice. Additionally, si-ERCC6 injection significantly decreased the apoptotic levels in the damaged spinal cord. This study also investigated the significantly enhanced apoptosis in LPS-exposed BV2 cells (Figures 3C,D). Nevertheless, si-ERCC6 treatment markedly alleviated apoptotic levels in LPS-exposed BV2 cells. The apoptotic proteins were further measured in spinal cord tissues. We noted that Bax and cleaved caspase-3 expression were markedly elevated as well as Bcl-2 was lowly expressed in the damaged spinal cord relative to sham operation mice (Figures 3E–H). As expected, si-ERCC6 administration remarkably reduced the expression of Bax and cleaved caspase-3 expression as well as enhanced the expression of Bcl-2 in the damaged spinal cord. Altogether, ERCC6 deficiency decreased apoptosis both in the damaged spinal cord and LPS-exposed mouse microglia cells.

### ERCC6 Knockdown Alleviates Inflammation in the Spinal Cord of ICI Mice

**FIGURE 4:**
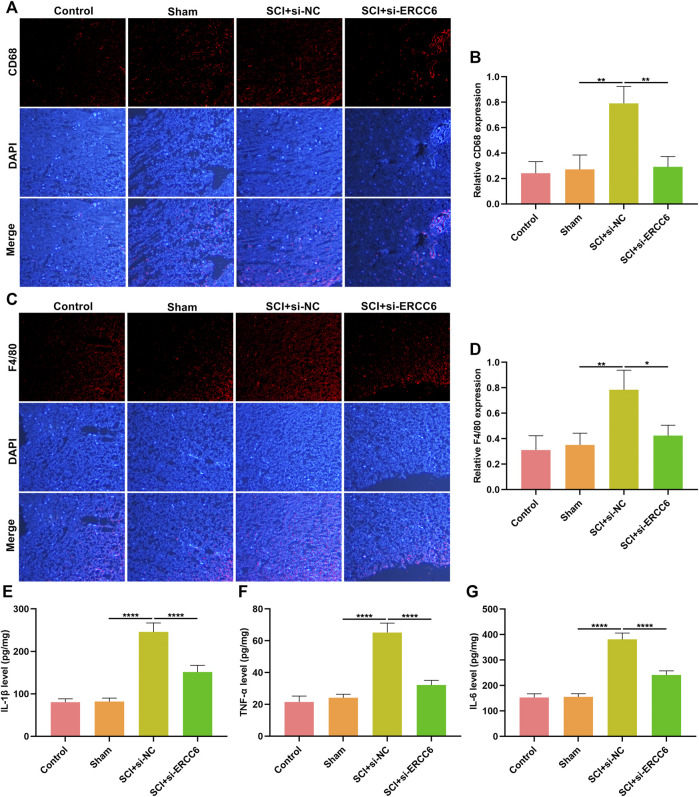
ERCC6 knockdown alleviates inflammation in the spinal cord of ICI mice. **(A,B)** Immunofluorescence staining of CD68 expression in the damaged spinal cord of mouse models injected by si-ERCC6 or si-NC as well as control and sham operation mice (scale bar, 50 μm). **(C,D)** Immunofluorescence staining of F4/80 expression in the spinal cord of mouse models injected by si-ERCC6 or si-NC as well as control and sham operation mice (scale bar, 50 μm). **(E–G)** ELISA analysis of the levels of inflammatory factors containing **(E)** IL-1β, **(F)** TNF-α as well as **(G)** IL-6 in spinal cord of mouse models injected with si-ERCC6 or si-NC as well as control and sham operation mice (N = 8 mice in each group). *p*-values were calculated with the ANOVA test. *p-value < 0.05; **p-value < 0.01; ****p-value < 0.0001.

Excessive inflammatory response participates in SCI pathogenesis (Rong et al., 2019; Liu et al., 2020). Herein, we investigated whether ERCC6 modulated inflammatory response during SCI. Immunofluorescence staining showed that macrophage markers CD68 as well as F4/80 were remarkably highly expressed in the spinal cord tissues of ICI mice relative to sham operation mice (Figures 4A–D). Nevertheless, si-ERCC6 administration significantly decreased the expression of CD68 and F4/80 in the spinal cord tissues of ICI mice. The levels of inflammatory factors containing IL-1β, TNF-α, and IL-6 were significantly higher in the spinal cord of ICI mice relative to sham operation mice (Figures 4E–G). By contrast, ERCC6 knockdown remarkably decreased the levels of IL-1β, TNF-α, and IL-6 in the spinal cord of ICI mice. Collectively, the abovementioned data indicated that ERCC6 deficiency alleviated excessive inflammatory responses in the damaged spinal cord.

### ERCC6 Ablation Alleviates Astrocyte and Microglia Activation in the Damaged Spinal Cord

**FIGURE 5:**
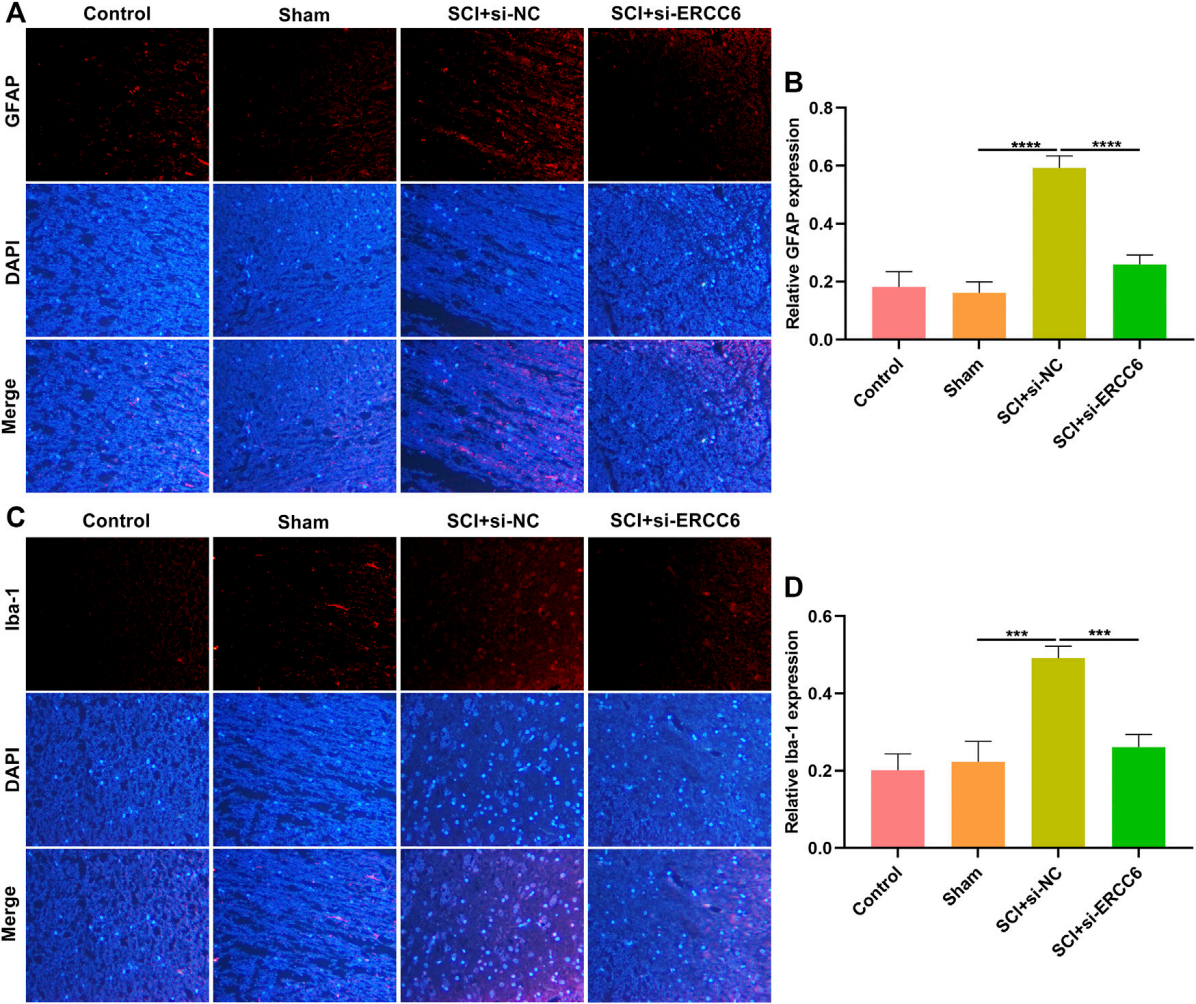
ERCC6 ablation alleviates astrocyte and microglia activation in the spinal cord of SCI mice. **(A,B)** Immunofluorescence staining of GFAP expression in the spinal cord tissues of SCI mice injected with si-ERCC6 or si-NC as well as control and sham operation mice (scale bar, 50 μm). **(C,D)** Immunofluorescence staining of Iba-1 expression in the spinal cord of mouse models injected by si-ERCC6 or si-NC as well as control and sham operation mice (scale bar, 50 μm; N = 8 mice in each group). *p*-values were calculated with the ANOVA test. ***p-value < 0.001; ****p-value < 0.0001.

Astrocyte and microglia activation were separately assessed through GFAP and Iba-1 immunofluorescence. As illustrated in Figures 5A,B, GFAP presented markedly higher expression in the damaged spinal cord relative to sham operation mice. Nevertheless, si-ERCC6 administration remarkably decreased GFAP expression in the damaged spinal cord, indicating that ERCC6 deficiency alleviated astrocyte activation in the damaged spinal cord. We also noted the significantly enhanced expression of Iba-1 in damaged spinal cord tissues, but si-ERCC6 administration decreased its expression (Figures 5C,D). This indicated that ERCC6 deficiency decreased microglia activation in the spinal cord of SCI mice.

### ERCC6 Ablation Ameliorates Cell Senescence in Damaged Spinal Cord and LPS-Induced Mouse Microglia Cells

**FIGURE 6:**
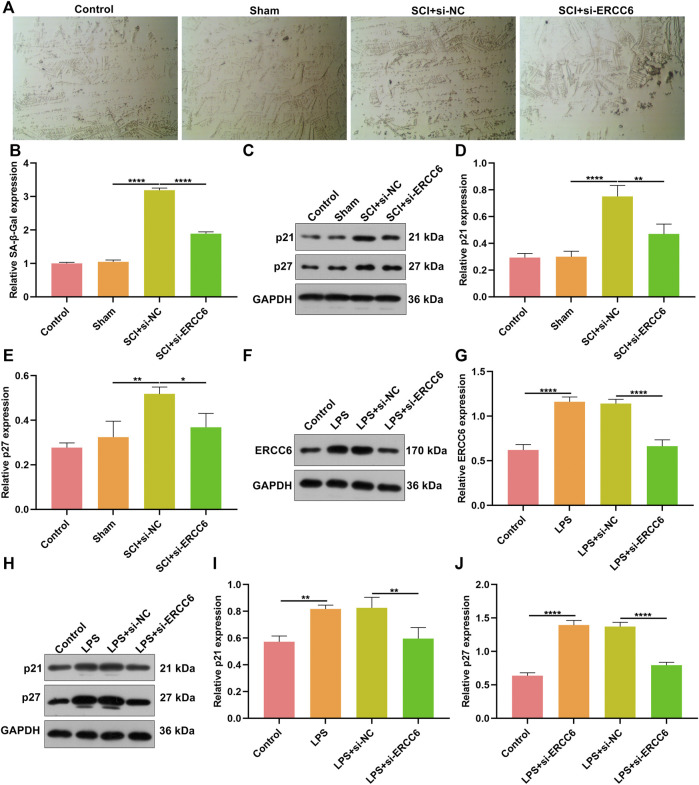
ERCC6 ablation ameliorates cell senescence in the spinal cord of SCI mice and LPS-induced mouse microglia cells. **(A,B)** SA-β-Gal staining of the spinal cord of mouse models injected by si-ERCC6 or si-NC as well as control and sham operation mice (scale bar, 50 μm; N = 8 mice in each group). **(C–E)** Western blotting for senescent markers p21 as well as p27 in the spinal cord tissues of SCI mice injected with si-ERCC6 or si-NC as well as control and sham operation mice (N = 8 mice in each group). **(F,G)** Western blotting analysis of ERCC6 expression in LPS-exposed BV2 mouse microglia cells treated with si-ERCC6 or si-NC (N = 3 mice in each group). **(H–J)** Western blotting analysis of the expression of senescent markers p21 and p27 in LPS-exposed BV2 mouse microglia cells treated with si-ERCC6 or si-NC (N = 3 mice in each group). *p*-values were calculated with the ANOVA test. *p-value < 0.05; **p-value < 0.01; ****p-value < 0.0001.

Further analysis was conducted for investigating the role of ERCC6 on neural senescence during SCI. Compared with sham operation mice, senescent cells were remarkably elevated in damaged spinal cord tissues (Figures 6A,B). However, si-ERCC6 treatment markedly decreased senescent cells in the damaged spinal cord. The expression of senescent markers p21 and p27 was also measured. Higher expression of p21 and p27 was found in spinal cord tissues of SCI mice relative to sham operation mice (Figures 6C–E). The expression of both was markedly reduced by si-ERCC6 administration in SCI mice. We also observed the role of ERCC6 on microglia senescence. First, our data affirmed that ERCC6 expression was remarkably enhanced in BV2 cells by LPS (Figures 6F,G). However, its expression was prominently decreased by si-ERCC6. As expected, there was a markedly increased expression of p21 and p27 in the LPS-exposed BV2 cells (Figures 6H–J). Their expression was alleviated by si-ERCC6 administration. Altogether, ERCC6 ablation enabled to ameliorate cell senescence both in the damaged spinal cord and LPS-induced microglia cells.

### ERCC6 Ablation Alleviates Oxidative Stress in the Spinal Cord of SCI Mice and LPS-Induced Microglia Cells

**FIGURE 7:**
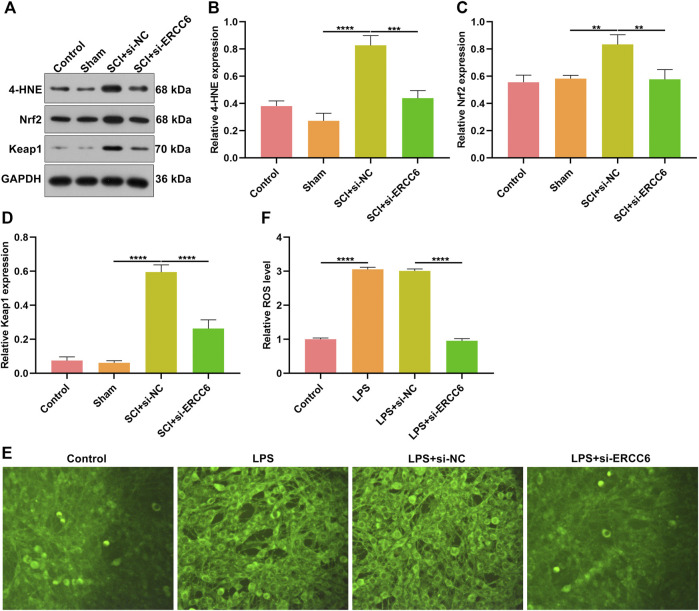
ERCC6 ablation alleviates oxidative stress in the spinal cord of SCI mice. **(A–D)** Western blotting analysis of the expression of oxidative stress markers 4-HNE, Nrf2, and Keap1 in the spinal cord of mouse models injected by si-ERCC6 or si-NC as well as control and sham operation mice (N = 8 mice in each group). **(E,F)** The levels of ROS fluorescence in LPS-exposed BV2 mouse microglia cells treated with si-ERCC6 or si-NC (scale bar, 50 μm; N = 3 mice in each group). *p*-values were calculated with the ANOVA test. **p-value < 0.01; ***p-value < 0.001; ****p-value < 0.0001.

Oxidative stress results in microglial and astrocyte activation, and promotes the release of inflammatory factors. Thus, the influence of ERCC6 on oxidative stress following SCI was further investigated. In comparison with sham operation mice, the expression of oxidative stress markers 4-HNE, Nrf2, and Keap1 was remarkably elevated in the damaged spinal cord (Figures 7A–D). However, si-ERCC6 administration markedly lowered the expression of 4-HNE, Nrf2, and Keap1 in the spinal cord of SCI mouse models. As illustrated in Figures 7E,F, there were significantly higher ROS levels in the LPS-induced BV2 cells relative to controls. Nevertheless, si-ERCC6 administration markedly lowered ROS accumulation in the LPS-induced BV2 cells. Hence, ERCC6 ablation alleviated oxidative stress in the spinal cord of SCI mice as well as LPS-induced BV2 cells.

## Discussion

SCI represents a severe neurological disease inducing neurological dysfunction and permanent injury (Wang et al., 2020). Hence, it is urgently required to develop novel effective therapeutic regimens against SCI-triggered neurological disorders and tissue damage. In the present study, ERCC6 blockage remarkably alleviated spinal cord damage by weakening apoptosis, inflammation, senescence, and oxidative stress both in the SCI mouse models and LPS-induced microglia cells. Thus, our findings provided a basis for future research on the targeted therapy following SCI regarding to ERCC6.

This research was the first evidence to demonstrate that ERCC6 expression was remarkably upregulated both in the damaged spinal cord of mouse models and LPS-induced microglia cells. Previously, ERCC6 upregulation was linked with aging-related diseases (age-related macular degeneration, *etc*.) (Baas et al., 2010). Following injection with ERCC6-siRNA-carrying lentivirus, neuronal damage of SCI mice was remarkably ameliorated. When the spinal cord is damaged, neuronal cellular deaths are key pathological events resulting in neurological deficiency (Wang et al., 2019; Abbaszadeh et al., 2020; Fang Wang et al., 2021). Meanwhile, apoptosis is a vital cell death process in SCI. ERCC6 blockage alleviated apoptotic levels in the damaged spinal cord of mouse models and LPS-induced microglia cells. The Bcl family is categorized as pro-apoptotic proteins (Bax, etc.) as well as anti-apoptotic proteins (Bcl-2, etc.). ERCC6 blockage upregulated Bax and cleaved caspase-3 as well as downregulated Bcl-2 in the spinal cord of SCI mouse models. Hence, ERCC6 blockage enabled to ameliorate the apoptotic process in the damaged spinal cord.

Neuroinflammation is a critical event during SCI and involves multiple cell types (Rong et al., 2019; Liu et al., 2020). Microglia cells are the main resident immune populations within the central nervous system (CNS), which can respond to stimuli in several minutes as well as colonize the damaged sites, thereby resulting in peripheral immune cell infiltrations (especially macrophages) (Lin et al., 2021). Excessive inflammation can promote secondary injury after SCI, accompanied by cascade excitement of cytokines (Lin et al., 2021). Our data demonstrated that ERCC6 blockage decreased the expression of macrophage markers CD68 as well as F4/80 in the damaged spinal cord. Moreover, its knockdown reduced the levels of IL-1β, TNF-α, and IL-6 in the damaged spinal cord of mouse models. The activation of GFAP-expressing astrocytes and Iba-1-expressing microglial cells are also key effectors of neuroinflammation (Bellver-Landete et al., 2019; Li et al., 2021). The expression of both was downregulated in the damaged spinal cord through blocking ERCC6. Thus, ERCC6 deficiency alleviated excessive inflammation in the damaged spinal cord.

Persistent senescent cells act as a determinant of the organ repair process (Wyss–Coray, 2016). In a previous study, cell senescence was induced in SCI-regenerating zebrafish and SCI-scarred mice (Paramos-de-Carvalho et al., 2021). Although the senescent cells induced in zebrafish were gradually eliminated, they were accumulated in mouse models over time. Using distinct aging agents, senescent cells in SCI mice were removed and the functions were recovered. The functional recovery was linked with the inhibition of fibrotic scars and inflammatory response (Paramos-de-Carvalho et al., 2021). Consistently, senescent cells were accumulated in the spinal cord of SCI mice. Thus, alleviating senescent cells is a prospective treatment regimen for SCI. Replicative senescence induced by replication-mediated DNA damage or shortened telomeres determines persistent DNA damage response as well as leads to the stabilization of transcription factor p53 and the expression of cyclin-dependent kinase inhibitor p21 (Xu et al., 2021). Higher expression of p21 and p27 was found in the spinal cord of SCI mice as well as LPS-induced microglia cells, indicating replicative senescence accumulation during SCI. ERCC6 promoter downregulation through histone H3 hypoacetylation blocks p21-independent replicative senescence (Crochemore et al., 2019). Herein, we noted that ERCC6 deficiency alleviated senescent cells as well as decreased the expression of p21 and p27 in the spinal cord of SCI mice and LPS-induced microglia cells. Hence, targeting ERCC6 might enable to ameliorate cell senescence in SCI.

Oxidative stress plays a destructive role during SCI, which generates ROS in the spinal cord to destroy protein, lipid, or DNA (Li et al., 2019). ROS accumulation enhances the demand for ascorbic acid as well as changes the capacity of antioxidant enzymes (Liu et al., 2020). Alleviating excessive oxidative stress can improve the locomotor functional recovery following SCI (Qian et al., 2019; Ungerer et al., 2020). Evidence suggests that ERCC6 enables to trigger oxidative DNA damage through recruiting XRCC1 (Menoni et al., 2018). Moreover, it mediates the chromatin structure as well as coordinates gene expression in response to oxidative stress (Lake et al., 2016). The Nrf2 signaling pathway represents the major reason why cells resist oxidative stress (Rao et al., 2019). In SCI mice, ERCC6 blockage downregulated the expression of oxidative stress markers 4-HNE, Nrf2, and Keap1 as well as excessive ROS accumulation in the LPS-induced BV2 cells. Hence, ERCC6 ablation ameliorated oxidative stress in the damaged spinal cord. However, several limitations should be pointed out. First, more experiments should be carried out to investigate the roles of ERCC6 on the recovery of motor function after SCI. Second, the molecular mechanisms involving ERCC6 in SCI should be further validated through *in vitro* and *in vivo* experiments.

## Conclusion

Altogether, ERCC6 expression presented remarkable upregulation both in damaged spinal cord of mouse models and LPS-induced microglia cell models. Its deficiency enabled to alleviate diverse physiological processes (apoptosis, inflammation, senescence, oxidative stress, etc.). Our findings implied that ERCC6 acted as a prospective therapeutic target against SCI.

## Data Availability

The original contributions presented in the study are included in the article/Supplementary Material, further inquiries can be directed to the corresponding authors.

## Abbreviations

SCI, spinal cord injury; ERCC6, excision repair cross-complementing group 6; LPS, lipopolysaccharide; siRNAs, small interfering RNAs; RT-qPCR, real-time quantitative PCR; TUNEL, terminal-deoxynucleoitidyl transferase mediated nick end labeling; ELISA, enzyme-linked immunosorbent assay; IL-1β, interleukin-1β; TNF-α, tumor necrosis factor-α; SA-β-Gal, senescence-associated β-galactosidase; ROS, reactive oxygen species; DCFH-DA, dichloro-dihydro-fluorescein diacetate.
